# Improving the use of research evidence in guideline development: 7. Deciding what evidence to include

**DOI:** 10.1186/1478-4505-4-19

**Published:** 2006-12-01

**Authors:** Andrew D Oxman, Holger J Schünemann, Atle Fretheim

**Affiliations:** 1 Norwegian Knowledge Centre for the Health Services, P.O. Box 7004, St. Olavs plass, N-0130 Oslo, Norway; 2 INFORMA, S.C. Epidemiologia, Istitituto Regina Elena, Via Elio Chianesi 53, 00144 Rome, Italy; 3 Norwegian Knowledge Centre for the Health Services, P.O. Box 7004, St. Olavs plass, N-0130 Oslo, Norway

## Abstract

**Background:**

The World Health Organization (WHO), like many other organisations around the world, has recognised the need to use more rigorous processes to ensure that health care recommendations are informed by the best available research evidence. This is the seventh of a series of 16 reviews that have been prepared as background for advice from the WHO Advisory Committee on Health Research to WHO on how to achieve this.

**Objectives:**

We reviewed the literature on what constitutes "evidence" in guidelines and recommendations.

**Methods:**

We searched PubMed and three databases of methodological studies for existing systematic reviews and relevant methodological research. We did not conduct systematic reviews ourselves. Our conclusions are based on the available evidence, consideration of what WHO and other organisations are doing and logical arguments.

**Key question and answers:**

We found several systematic reviews that compared the findings of observational studies with randomised trials, a systematic review of methods for evaluating bias in non-randomised trials and several descriptive studies of methods used in systematic reviews of population interventions and harmful effects.

What types of evidence should be used to address different types of questions?

• The most important type of evidence for informing global recommendations is evidence of the effects of the options (interventions or actions) that are considered in a recommendation. This evidence is essential, but not sufficient for making recommendations about what to do. Other types of required evidence are largely context specific.

• The study designs to be included in a review should be dictated by the interventions and outcomes being considered. A decision about how broad a range of study designs to consider should be made in relationship to the characteristics of the interventions being considered, what evidence is available, and the time and resources available.

• There is uncertainty regarding what study designs to include for some specific types of questions, particularly for questions regarding population interventions, harmful effects and interventions where there is only limited human evidence.

• Decisions about the range of study designs to include should be made explicitly.

• Great caution should be taken to avoid confusing a lack of evidence with evidence of no effect, and to acknowledge uncertainty.

• Expert opinion is not a type of study design and should not be used as evidence. The evidence (experience or observations) that is the basis of expert opinions should be identified and appraised in a systematic and transparent way.

## Background

The World Health Organization (WHO), like many other organisations around the world, has recognised the need to use more rigorous processes to ensure that health care recommendations are informed by the best available research evidence. This is the seventh of a series of 16 reviews that have been prepared as background for advice from the WHO Advisory Committee on Health Research to WHO on how to achieve this.

Recommendations about health care and about interventions or actions that affect health, such as social or environmental interventions, can be informed by a wide range of evidence including randomised trials, non-randomised comparative studies, descriptive studies, qualitative research, animal studies and laboratory studies. Discussions of evidence-informed policy and practice can generate debates regarding what constitutes 'evidence' [1]. A common understanding of evidence is that "evidence concerns facts (actual or asserted) intended for use in support of a conclusion" [1]. A fact, in turn, is something known by experience or observation. An important implication of this understanding of evidence is that evidence is used to support a conclusion; it is not the same as the conclusion. Evidence alone does not make decisions.

This understanding of what evidence is has several implications. Firstly, expert opinion is more than evidence. It combines facts, interpretation of those facts, and conclusions. There is evidence behind expert opinions. Expert opinion should be used appropriately by identifying the facts (experience or observations) that are the basis of the opinions and appraising the extent to which the facts support the conclusions [2].

Secondly, not all evidence is equally convincing. How convincing evidence is (for effects) should be based on criteria such as: What sort of observations? How well were they done? How consistent are they? How directly relevant are they? How many are there? How strong is an association?

Thirdly, judgements about how much confidence to place in different types of evidence (the 'quality' of the evidence) are made either implicitly or explicitly. It is better to make these judgements systematically and explicitly to help protect against errors, resolve disagreements, facilitate critical appraisal, and communicate information. This, in turn, requires explicit decisions about what types of evidence to consider at all.

Fourthly, all evidence is context sensitive, since observations are made in a specific context. A judgement always needs to be made about their applicability beyond that context. It is best to make judgements about applicability systematically and explicitly, for the same reasons that it is best to make judgements about the quality of the evidence systematically and explicitly.

Fifthly, global evidence (i.e. the best evidence from around the world) is the best starting point for judgements about effects, likely modifying factors, and (sometimes at least) resource utilisation. This argument is based on the understanding that all evidence is context sensitive to some extent and, therefore, indirect to some extent. Decisions based on a subset of observations are more prone to random errors [3], and judgements about whether to base a conclusion on a subset of observations are better informed if the overall observations (all of the relevant global evidence) are known [4].

Sixthly, local evidence (from the specific setting in which decisions and actions will be taken) is needed for most other judgements about what to do, including: the presence of modifying factors in specific settings, need (prevalence, baseline risk or status), values, costs and the availability of resources.

Recognising the need for both global evidence (of effects) and local evidence, it is important to be cautious about developing global recommendations. Nonetheless, global recommendations are valuable when different local conditions are not likely to lead to different decisions. When different conditions are likely to lead to different decisions, global frameworks for decisions are still important. These can reduce unnecessary duplication of efforts. They are particularly important to support low and middle-income countries, with limited resources to systematically develop guidelines, to make context specific decisions by providing the global evidence, a framework for decisions, and practical advice for incorporating local evidence.

WHO's focus is on global recommendations and supporting its member states to make well-informed decisions. The primary question that needs to be addressed in this context is:

• What types of study designs should be used to address different types of questions about the effects of the different options that are considered when making a recommendation?

We therefore have focused this review on questions about effects, recognising that there are parallel questions regarding what types of study designs should be used to address other questions. In addressing this question we have focused on the validity of different study designs, assuming that questions about the applicability of the results of studies to the specific questions of interest will be similar across different study designs. However, it is important to recognise that decisions about what study designs to include may also be influenced by the extent to which relevant studies are available that have used study designs that are most likely to provide valid results. That is, there may sometimes be a trade-off between including studies that are more likely to be valid and ones that are more likely to be directly relevant.

## What WHO is doing now

The Guidelines for WHO Guidelines (GWG) state: *"It is recommended that [a] systematic review be undertaken () *After the studies have been identified and critically appraised, and the evidence synthesised, evidence should be graded. All evidence, including that on safety, should be clearly laid out in an evidence table. Meta-analysis should be done when the data permit. The final results should be presented in a balance sheet" [5]. The GWG do not address the choice of study designs for different types of questions. In practice it is difficult to know what study designs are considered relevant for different types of WHO recommendations since few WHO guidelines have adhered to the GWG, few have included a systematic review, and many do not include references [6,7].

## What other organisations are doing

The U.S. Preventive Services Task Force has the following approach to determining what evidence is admissible:

*The topic team determines the bibliographic databases to be searched and the specific inclusion and exclusion criteria (i.e., admissible evidence) for the literature on each key question. Such criteria typically include study design, population studied, year of study, outcomes assessed, and length of follow-up. Topic teams specify criteria on a topic-by-topic basis rather than adhering to generic criteria. If high-quality evidence is available, the topic teams may exclude lower-quality studies. Conversely, if higher-quality evidence is lacking, the teams may examine lower-quality evidence*.

*If a search finds a well-performed systematic review that directly addresses the literature on a key question through a given date, the topic team may use this review to capture the literature for those dates. The team can then restrict its own search to dates not covered by the existing systematic review*.

*The topic team documents these strategies for sharpening focus – the analytic framework, key questions, and criteria for admissible evidence – in an initial work plan. This work plan is presented to the Task Force at its first meeting after the topic has been assigned, allowing the Task Force the opportunity to modify the direction and scope of the review, as needed *[8].

This approach is consistent with other guidance for systematic reviews, such as those of the Cochrane Health Promotion and Public Health Task Force, which recommends that:*"The study designs to be included in a public health review should be dictated by the interventions being reviewed (methodological appropriateness), and not vice versa" *[9]. There is also general, although not unanimous, agreement that the inclusion criteria for a systematic review should specify the study designs that are acceptable for a specific question [10]. However, there are important differences in both guidance and practice with respect to "how low" reviewers should go in deciding what evidence to include [11]. This question is particularly relevant for questions about the effects of population interventions (public health, health promotion, health systems and social interventions) and for evidence of harmful effects [10-20].

The Cochrane Handbook for Systematic Reviews of Interventions takes a relatively cautious approach: "*The more restrictive authors are in matching questions to particular aspects of design, the less likely they are to find data specific to the restricted question. However, reviewing studies that are unlikely to provide reliable data with which to answer the question is a poor use of time and can result in misleading conclusions." *[21] Because Cochrane reviews address questions about the effects of health care, they focus primarily on randomised trials. The Handbook suggests being cautious of including non-randomised studies because of the risk of biased results; the additional work required to identify and appraise non-randomised studies and keep a review up-to-date; and the risk of publication bias. It concludes: "*While attention to the risk of bias should guide decisions about what types of study designs to include in a review, individual authors and Collaborative Review Groups must decide what types of studies are best suited to specific questions."*

Within the Cochrane Collaboration, several groups have recommended inclusion of a broader range of study designs for health systems and public health interventions and for assessing harmful effects of clinical interventions. The Cochrane Effective Practice and Organisation of Care Group (EPOC) argues that: *While cluster randomised trials are the most robust design for quality improvement strategies, some strategies may not be amenable to randomisation – for example, mass media campaigns. Under these circumstances, reviewers may choose to include other designs including quasi-experimental designs. If a review includes quasi-experimental studies – for example, interrupted time series designs for evaluating mass media campaigns, the reviewers need to recognise the weaknesses of such designs and be cautious of over-interpreting the results of such studies. Within EPOC, reviewers can include randomised trials, controlled before and after studies, and interrupted time series *[17].

The Guidelines for Systematic Reviews of Health Promotion and Public Health Interventions Taskforce suggests including a still broader range of study designs: *"A wide variety of study designs may be used in the evaluation of public health activities, ranging from randomized controlled trials (RCTs) to case studies, with no single method being able to answer all relevant questions about the effectiveness of all public health interventions." *[9]

The Cochrane Adverse Effects Subgroup identifies three possible approaches for incorporating adverse effect data in a review and summarises the advantages and disadvantages of each of these approaches as summarised in Table 1[18,19].

**Table 1:** Pros and cons of different approaches for incorporating adverse effect data in a systematic review*

**Method**	**Look in the trials/studies included in the systematic review of benefit.**	**Look in all retrieved trials/studies of that intervention, even in those excluded from the analysis of benefit**	**Look for studies that specifically evaluate adverse effects of the intervention**
Protocol	Should usually be the minimum recommendation	Studies rejected from analysis of benefit (e.g. because beneficial outcomes are measured in a different way, which cannot be combined with other studies), may be included to allow adverse effect data collection. Two sets of inclusion criteria will be needed – for benefit, and for adverse effects	Design separate strategy to identify studies that report adverse effects, including those that do not look at beneficial effects.
			Might amount to a separate review nested within a traditional Cochrane review
Pros	Less demanding on time and resources	More comprehensive than just looking at included trials	Most comprehensive
	Does not require new literature search strategy	Can potentially cover a more representative group of patients	May be able to evaluate rare, or long-term, or previously unrecognized adverse effects
Cons	Data may be very limited and biased towards common, short-term harms	Relatively time consuming as full-text articles of all potentially relevant studies need checking Data may be limited to well-recognized and commonly seen adverse effects.	Time and resource intensive
		Benefit and harm cannot be compared directly as the data come from different sources	Special techniques required in synthesizing data from a diverse range of sources
			Increased quantity of data but greater risk of biased and poor quality data
			Benefit and harm cannot be compared directly as the data come from different sources.

*Copied from reference [18].

The U.K. NHS Centre for Reviews and Dissemination provides the following guidance: *"The inclusion criterion specifying the type of study design stems from the desire to base reviews on the highest quality evidence. There are several areas of health care which have not been evaluated with methodologically sound studies. In this situation, studies of methodologically lower quality may have to be included. Here it is important to note that the preference for one or another study design should depend on the nature of questions raised in the review. Inevitably the decisions regarding inclusion based on study design will also depend on the availability of suitable study designs in the literature." *[22]

We are not aware of any specific guidance for what study designs to include for non-human studies, although some recommendations rely on animal and in vitro studies. For example, treatment recommendations for emerging diseases, such as SARS or avian influenza (H5N1), for which case reports may be the only human studies that are available, may be based on a combination of indirect human evidence (from the treatment of other similar diseases), case reports, animal studies and in vitro studies. In general, the same principles that apply to human studies can be applied to animal and in vitro studies [23].

The Guide to Community Preventive Services uses data from comparative studies – those that compare outcomes among a group exposed to the intervention versus outcomes in a concurrent or historical group that was not exposed or was less exposed – to answer questions about whether interventions are effective [24]. All comparative studies are included in its reviews, assessed for their design suitability and threats to internal and external validity, and assessed for potential effects of study design and execution on results.

The Campbell Collaboration does not provide specific guidance on what study designs should be used to address different types of questions related to the effects of interventions in the social, behavioral and educational arenas [25].

## Methods

The methods used to prepare this review are described in the introduction to this series [26]. Briefly, the key questions addressed in this paper were vetted amongst the authors and the ACHR Subcommittee on the Use of Research Evidence (SURE). We did not conduct a full systematic review. We searched PubMed and three databases of methodological studies (the Cochrane Methodology Register, the US National Guideline Clearinghouse, and the Guidelines International Network for existing systematic reviews and relevant methodological research that address these questions. The answers to the questions are our conclusions based on the available evidence, consideration of what WHO and other organisations are doing, and logical arguments.

For this review we searched PubMed using (clinical practice guidelines or public health guidelines or systematic reviews) and (study designs) and related articles for references. We searched the Cochrane Methodology Register using the key word study design, and we checked the reference lists of the reports that we retrieved. The searches were conducted in February and March 2006.

## Findings

We found several systematic reviews that compared the findings of observational studies with randomised trials [27-33], and a systematic review of methods for evaluating bias in non-randomised trials [34]. We also found several descriptive studies of methods used in systematic reviews of population interventions and harmful effects.

Systematic reviews of the results of randomised trials compared with observational studies have differed in the methods they have used, and, to some extent, in their conclusions, but have generally found that it is not possible to predict differences in the size, or even the direction, of estimates of treatment effects for the same intervention when it is generated in randomized and non-randomized studies. However, especially in the more recent reports [30-33], there is the suggestion that these disparities decrease when investigators have controlled for known confounders (between risk/responsiveness and treatment).

The review of methods for evaluating bias in non-randomised trials found six tools that were thought to be suitable for use in systematic reviews [34]. Their review of 511 systematic reviews that included non-randomised studies found that only 169 (33%) assessed study quality. A more recent survey of methods used in systematic reviews of adverse effects found that although more than three quarters (185/243) reviews sought to include data from sources other than randomised controlled trials, fewer than half (106/256) assessed the quality of the studies that were included [35].

A study that considered the potential of randomised trials to provide evidence on specific harms found that of 1727 Cochrane reviews, only 138 included evidence on ≥ 4000 subjects. Of these only 25 (18%) had eligible data on adverse events, while 77 had no harms data, and 36 had data on harms that were non-specific or pertained to < 4000 subjects [17]. Thus, while systematic reviews of randomised trials can provide useful information on adverse effects of clinical interventions, the reporting of adverse effects in both randomised trials and systematic reviews needs to be improved.

Descriptive reports of reviews of harmful effects have found that a significant investment of effort failed to yield significant new information [18,19,36]. Authors of reviews of social interventions, on the other hand, have argued that restricting the study designs that are included in a review may reduce the value of the review and reinforce the "inverse evidence law" whereby the least is known abut the effects of interventions most likely to influence whole populations. However, this argument relates more to the importance of mapping out the available evidence than to producing reliable estimates of the effects of interventions [11].

## Discussion

While there is broad agreement that the study designs to be included in a review should be dictated by the interventions being reviewed, there is uncertainty regarding what study designs to include for some specific types of questions. For any question, as the range of study designs that are included is broadened, an increasing amount of work is required to derive decreasingly reliable estimates of the effects of interventions. A decision about how broad a range of study designs to consider must be made in relationship to the characteristics of the interventions, what evidence is available, and the time and resources available.

For any question there is a cut-off point beyond which broadening the types of studies that are considered requires a substantial investment of effort that will not yield additional information that will inform decisions in a meaningful way. In many cases, it is likely to be prudent to acknowledge the limits of what is known from a restricted range of study designs, rather than to invest additional resources that are unlikely to do more than confirm the limits of what is known. Whatever decision is taken about the range of study designs to include should be made explicit, and great caution should be taken to avoid confusing a lack of evidence with evidence of no effect.

## Further work

There is a rapidly growing number of reviews and studies comparing the results of different study designs. High priority should be given to generating and periodically updating a common data set of studies to update and reconcile different conclusions among these reviews. Priority should also be given to broadening the scope of these comparisons to include a wider range of questions and a wider range of study designs, including animal and laboratory studies. Additional studies, and systematic reviews of studies, that more rigorously assess the added cost and value of including broader ranges of study designs would help to inform decisions about when it is likely to be important and worthwhile to use more diverse types of study designs. There is a need to develop more detailed guidance regarding decisions for which study designs to include for different types of questions for incorporation in the Guidelines for WHO Guidelines. This guidance, which is particularly needed for harms and interventions targeted at populations, should be based on both empirical evidence and conceptual arguments.

## Competing interests

ADO and AF work for the Norwegian Knowledge Centre forthe Health Services, an agency funded by the Norwegian government that produces systematic reviews and health technology assessments. All three authors are contributors to the Cochrane Collaboration. ADO and HJS are members of the GRADE Working Group. HJS is documents editor and chair of the documents development and implementation committee for the American Thoracic Society and senior editor of the American College of Chest Physicians' Antithrombotic and Thrombolytic Therapy Guidelines.

## Authors' contributions

ADO prepared the first draft of this review. HJS and AF contributed to drafting and revising it.
